# Dietary Trace Elements and Arsenic Species in Rice: A Study of Samples from Croatian Supermarkets

**DOI:** 10.3390/foods14132261

**Published:** 2025-06-26

**Authors:** Ivana Rumora Samarin, Antonija Sulimanec, Tatjana Orct, Anica Benutić, Bernardo Marciuš, Karla Tomljanović, Jasna Jurasović

**Affiliations:** 1 Faculty of Food Technology and Biotechnology, University of Zagreb, Pierottijeva 6, 10000 Zagreb, Croatia; karla.tomljanovic1@gmail.com; 2 Institute for Medical Research and Occupational Health, Ksaverska Cesta 2, 10000 Zagreb, Croatiajurasovic@imi.hr (J.J.); 3 Croatian Institute of Public Health, Rockefellerova 7, 10000 Zagreb, Croatia; anica.benutic@hzjz.hr (A.B.); bernardo.marcius@hzjz.hr (B.M.)

**Keywords:** essential elements, food analysis, minerals, rice (*Oryza sativa* L.)

## Abstract

Rice (*Oryza sativa* L.) is a vital staple food and an important source of energy and macro- and micronutrients for billions of people. However, rice can accumulate undesirable levels of toxic trace elements, especially inorganic arsenic, which may pose a health risk. This study aimed to determine the concentrations of 29 essential and toxic elements and the fractions of four As species in 58 rice samples purchased in Croatian supermarkets. In addition, the influence of rice variety, cultivation methods, and origin on the composition of trace elements was analysed. The elements were quantified using inductively coupled plasma mass spectrometry (ICP-MS), and As species were quantified using high-performance liquid chromatography (HPLC) coupled with ICP-MS. Organic brown rice had higher concentrations of essential trace elements (Se, Zn, Cu, Fe, Mn, Co, Cr) than white rice, with organic brown rice containing more essential elements than conventionally grown rice. The average total arsenic concentration (tAs) across all samples was 142 ± 57 µg/kg, with brown, conventionally grown rice containing a higher amount. Arsenite was the predominant arsenic species. Regional differences in As and Se concentrations were observed. These results emphasize the complex relationship between trace elements in rice and their potential impacts on health.

## 1. Introduction

Rice (*Oryza sativa* L.) is one of the most widely consumed cereals in the world, especially in Asian countries. Currently, more than 538.9 million tons of rice are produced annually worldwide [1], with China, India, and Bangladesh being the largest producers [2,3]. Due to its high carbohydrate content (mainly starch), rice is not only an important source of energy but also a good source of micronutrients, including minerals [4,5,6,7]. Moreover, rice is increasingly chosen as a suitable carrier for delivering micronutrients [8]. However, despite its valuable nutritional composition, rice can contain some toxic elements in undesirable amounts, especially when grown on contaminated soils.

Arsenic (As), together with its inorganic species (iAs), arsenite (As^III^), and arsenate (As^v^), is categorized in “Group 1” of human carcinogens [9]. Long-term dietary exposure to iAs has generally been associated with an increased risk of bladder, lung, and skin cancer as well as cardiovascular diseases and diabetes mellitus [10]. Organic As species (oAs), monomethylarsonic (MMA) and dimethylarsinic acid (DMA) have a much lower toxicity than iAs and are the predominant As species in food of marine origin after the non-toxic arsenobetaine (AsB) [11,12]. Unfortunately, iAs is a predominant As form in rice, with a mean proportion of about 55% of total As (tAs) concentrations (range: 11–91%) [13]. Increased As accumulation in rice is either natural, as a result of soil biogeochemistry, or anthropogenic, e.g., due to industrial activities, combustion of fossil fuels, application of arsenic-containing pesticides/insecticides, fertilization, or irrigation with As-contaminated groundwater [14,15].

Among cereals, rice is the most efficient in the uptake of As into the grain [16], which is mainly due to its high uptake under reduced environmental conditions [17]. An accumulation of As in rice can disturb the balance of microelements [15]. Several studies have found that higher levels of As in rice are generally associated with lower levels of Se. Selenium reduces As toxicity through the formation of a complex that increases the excretion of both elements or through its antioxidant effect [18]. On the other hand, some microelements such as P, S, and N can modulate the biogeochemical cycle of As by either reducing the uptake and accumulation of As in the grain or supporting the oxidation of As^III^ to As^V^ [19]. In addition to the genotype, cultivation method, and environmental effects (biogeochemistry, climate, season) [2], food processing also affects the element composition of the rice grain. For example, post-harvest processes can subsequently reduce (e.g., polishing) or increase (e.g., parboiling) the content of essential and toxic elements in the final rice product [20,21,22]. Assuming that the water used is potable (i.e., 0.01 ppm iAs according to WHO recommendations), washing or soaking the rice removes up to 40% of iAs, and some cooking treatments even more (e.g., parboiling with the absorption method reduces up to 54 to 73% of iAs) [23]. An increasing number of studies are focusing on the quantification of elements in rice, primary As and its species (iAs and oAs), to ensure the safety and quality of this cereal for consumers.

Recently, the European Food Safety Authority (EFSA) [8] stated that rice and rice-based products are among the main contributors to dietary iAs intake among the EU population, alongside drinking water. Infants, toddlers, and young children were identified as the most vulnerable population group, with an average iAs intake of 0.30 to 0.61 µg/kg body weight per day. In adults, the estimated mean dietary iAs exposure was between 0.03 and 0.15 µg/kg body weight per day.

In Croatia, white and brown rice is available on the market and can be organically or conventionally grown. The labels on the rice products often contain additional information on grain size, origin, and cooking instructions.

Several studies in neighbouring countries have documented the As content in rice. A comprehensive study on Italian rice (*n* = 101) found tAs concentrations between 180 and 280 µg/kg and inorganic As between 80 and 110 µg/kg, with differences depending on geographical origin and type of grain processing [24]. In Slovenia, a market study with 50 rice samples (white, basmati, parboiled, and brown rice) showed an average tAs content of 157 ± 60 µg/kg and an inorganic As content of 90 ± 35 µg/kg, with significantly higher levels in brown and parboiled rice compared to basmati [5]. These results emphasize similar distribution patterns and reinforce the concern about rice consumption in this region.

This study aimed to determine the concentrations of elements of nutritional and toxicological interest (Mg, P, K, Ca, Fe, Mn, Co, Cu, Zn, Se, Mo, tAs, and others) and the fractions of four As species from the Croatian market. In addition, the trace element concentrations were compared according to rice variety, cultivation method, and origin, which can be used for a risk–benefit assessment related to rice consumption.

To our knowledge, this is the first comprehensive study to assess the elemental composition and arsenic speciation in rice samples available on the Croatian market, which could provide comparative insights relevant to EU food safety monitoring programs. The analysis of both inorganic and organic arsenic species, as well as essential and toxic elements, provides a comprehensive overview of the nutritional and safety profile of rice, which is essential for informed consumer choices and regulatory decisions.

## 2. Materials and Methods

### 2.1. Samples

A total of 58 rice samples were purchased in various stores (markets, supermarkets, health food stores) located in the capital city of Zagreb, Croatia. The samples were categorized into three groups: white (*n* = 31), parboiled (*n* = 7), and brown rice (*n* = 20) (Supplementary Materials, Table S1). In short, 35 samples originated from Italy, 13 from other European Union (EU) countries (Belgium, Germany, France, and unspecified EU countries), 12 from Asia (India, Pakistan, Thailand, and Cambodia), and 3 samples were of unknown origin. According to the type of rice cultivation method, 20 samples came from organic and 38 samples from conventional cultivation. All samples of parboiled rice were conventionally produced.

### 2.2. Sample Preparation for as Speciation and Quantification

First, all air-dried (as sold) rice samples were milled and homogenized using the B-400 blender (BÜCHI, Labortechnik AG, Flawil, Switzerland). Prior to speciation, As was extracted from the samples (~1 g rice powder) with 10 mL 0.28 M nitric acid (HNO_3_) in a water bath at 95 °C for 90 min with continuous shaking. After cooling to room temperature, the extracts were diluted with 6.7 g ultrapure water and then centrifuged (4000 rpm for 10 min) and filtered (0.45 μm PTFE Luer Lock syringe membrane filter). Prior to analysis, the pH of samples was adjusted using a pH adjustment solution (NH_4_OH in H_3_PO_4_ mobile phase, pH 9.85 ± 0.05) at a 1:2 ratio directly in the HPLC vial. Arsenic species were quantified using high-performance liquid chromatography (HPLC) (1260 Infinity II LC System, Agilent Technologies, Santa Clara, CA, USA) coupled to inductively coupled plasma mass spectrometry (ICP-MS) (7900, Agilent Technologies, Inc., Santa Clara, CA, USA). Chromatographic separation was performed on an anion exchange column (PRP-X100, 10 µm, 250 × 4.1 mm, Hamilton^®^, Hamilton, Bonaduz, Switzerland). The mobile phase consisted of 20 mM phosphoric acid (H_3_PO_4_) and 2% methanol (CH_3_OH) and was adjusted to pH 6.5 with 28% ammonia. A standard reference material, RM 1568b rice flour (NIST, Gaithersburg, MD, USA), was used as a quality control, with an average recovery rate of 103%. Five-point calibrations (of 1.0, 2.0, 3.0, 4.0, and 5.0 µg/L) were performed with As species stock solutions containing As^III^, As^V^, MMA, DMA, and AsB. The limits of detection (LoDs) for the As species analysed are summarized in Table 1. All As concentrations are expressed in µg/kg on a dry weight basis.

### 2.3. Multielement Analysis—Sample Digestion and Quantification

Rice homogenates were digested in an UltraCLAVE IV microwave digestion unit (Milestone, Sorisole, Italy). Approximately 0.200 g of the sample was placed in a quartz tube and mixed with a solution containing 2 mL of purified HNO_3_ (65% p.a., Merck, Darmstadt, Germany) and 3 mL of ultrapure water (BarnsteadTMSmart2Pure 6 UV/UF, Thermo Scientific, Langenselbold, Germany). Digestion was performed according to the temperature program shown in Supplementary Materials, Table S2. All samples were prepared and digested in duplicate, as was SRM 1568b rice flour (NIST, Gaithersburg, MD, USA) (*n* = 4), which was used as the reference material. After digestion and cooling to room temperature, the samples were adjusted to 6 g with ultrapure water.

Element concentrations (Mg, P, K, Ca, Fe, Mn, Co, Cu, Zn, Se, Mo, tAs, and others) in the digested rice samples were analysed using the 7500cx ICP-MS (Agilent Technologies, Tokyo, Japan) under the following conditions: MicroMist nebulizer; Scott-type quartz spray chamber cooled at 2 °C; plasma gas flow rate of 15 mL/min; carrier gas flow rate of 1.03 mL/min; make-up gas flow rate of 0.1 mL/min; RF power 1550 W; reaction cell gas: helium, hydrogen, or no gas. Prior to analysis, all samples were diluted 20-fold with a solution containing 1% (*v*/*v*) HNO_3_ and 3 µg/L internal standards (Ge, Rh, Tb, Lu, and Ir) (SCP Science, Baie-d’Urfé, QC, Canada).

All samples were analysed in triplicate. Element concentrations were quantified using a matrix-matched calibration. The accuracy was within 5% of the certified values in SRM 1568b for all analysed elements except for K and Co (≥10%). All element concentrations are expressed in mg/kg on a dry weight basis.

### 2.4. Statistical Analysis

The R programming language (v 4.5) and Rstudio (v 2024.12.2, Posit Software) were used to analyse the data. The results were expressed as arithmetic mean with standard deviation and total range in brackets. The Kruskal–Wallis test and post-hoc pairwise multiple comparison of mean ranks Dunn’s test were used to test for differences between groups (white, parboiled, and brown rice). Pearson’s correlation (r) was used to test the relationships between the elements. Statistical significance was set at 5% (*p* < 0.05).

## 3. Results

### 3.1. Arsenic in Rice

The results of tAs and arsenic species concentrations (As^III^, As^V^, MMA, DMA, including the sum of As^III^ and As^V^ as inorganic fraction) for the total sample and the distribution among the rice varieties are shown in Table 1.

**Figure 1:**
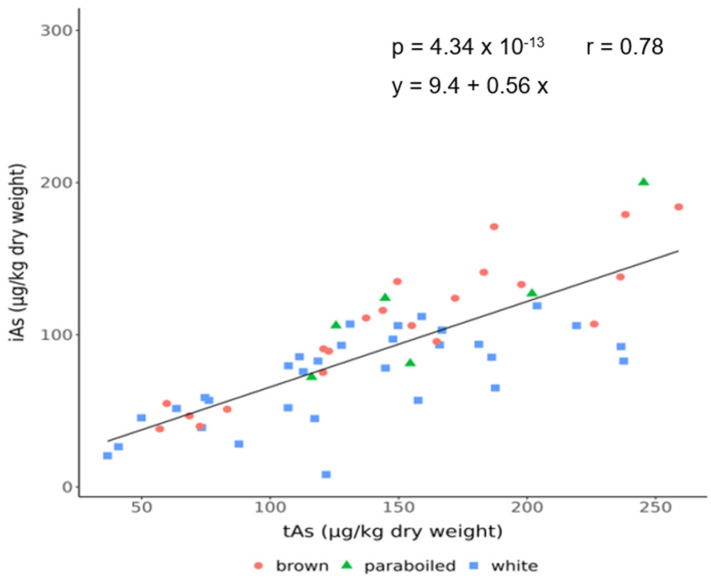
Relationship between total arsenic (tAs) and the fraction of inorganic arsenic species (iAs; as a sum of As^III^ and As^V^) in analysed rice samples.

Figure 1 shows the ratio between tAs and iAs in the analysed rice samples (n = 58) from the Croatian market. It can be seen that iAs is positively correlated with tAs, and the same correlation is found when the subgroups are considered.

We found no differences in the concentrations of tAs and all analysed arsenic species between samples of non-organically and organically grown rice.

**Table 1:** Total arsenic (tAs) and arsenic species concentrations (As^III^, As^V^, MMA, DMA, and the inorganic fraction as a sum of As^III^ and As^V^; in µg/kg dry weight) in white, parboiled, and brown rice available on the Croatian market and in standard reference material, SRM 1568b rice flour (NIST, Gaithersburg, MD, USA).

Arsenic (As) (µg/kg d.wt.)	All Rice (*n* = 58)	White Rice (*n* = 31)	Parboiled Rice (*n* = 7)	Brown Rice (*n* = 20)	SRM 1568b (*n* = 4)
	Mean ± SD (range)	Mean ± SD (range)	Mean ± SD (range)	Mean ± SD (range)	Mean ± SD [Mean ± 95% CI]
Total As	142.2 ± 57.1 (36.7–259.0)	132.3 ± 55.1 (36.7–237.6)	164.7 ± 49.6 (116.1–245.3)	150.3 ± 61.2 (57.0–259.0)	283 ± 1 [285 ± 14]
Inorganic As					
As^III^	55.5 ± 26.1 (6.6–161.0)	49.6 ± 22.5 (6.6–85.8)	63.8 ± 11.4 (48.1–80.2)	61.7 ± 32.1 (7.0–161.0)	41.6 ± 6.5 /
As^V^	33.9 ± 24.0 (<LoD–120.1)	22.9 ± 13.4 ^a^ (<LoD–52.3)	54.5 ± 35.7 ^b^ (24.0–120.1)	44.3 ± 25.0 ^b^ (7.3–83.8)	58.4 ± 10.1 /
As^III^+As^V^	89.3 ± 41.0 (8.1–200.0)	72.2 ± 29.5 ^a^ (8.1–119.0)	118.3 ± 45.7 ^b^ (72.1–200.0)	106.0 ± 44.5 ^b^ (38.1–184.0)	100 ± 5 [92 ± 10]
Organic As					
MMA	<LoD	<LoD	<LoD	<LoD	12.0 ± 1.5 [11.6 ± 3.5]
DMA	33.0 ± 28.1 (3.5–170.0)	38.7 ± 35.2 (3.5–170.0)	29.2 ± 17.1 (3.5–54.0)	25.6 ± 14.0 (3.5–52.8)	175 ± 17 [180 ± 12]

LoD—limit of detection (in µg/kg): tAs: 0.006; As^III^: 3.2; As^V^: 3.4; MMA: 5.0; DMA: 3.3. Horizontally, different lowercase letters denote significant differences (at *p* < 0.05) tested by Kruskal–Wallis ANOVA and post-hoc pairwise multiple comparison of mean ranks for three groups (white, parboiled, and brown rice) using Dunn’s test.

We estimate the intake of iAs from one serving portion of different rice types available on the Croatian market for an adult person and assess potential risk from the associated dietary exposure (Table 2).

### 3.2. Multielement Analysis—Macro and Trace Elements in Rice

The elemental composition data of the digested rice samples, quantified by ICP-MS for the total sample and separately by rice type, are shown in Table 3 and Appendix A, in Table A1.

**Figure 2:**
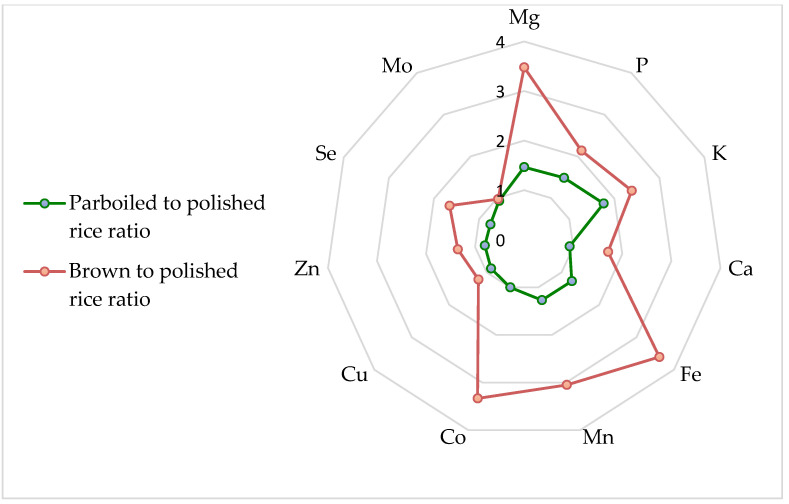
Average ratio of element concentration in rice for brown and parboiled rice compared to white rice; a value above 1 means an enrichment of elements compared to white rice.

Based on the results presented in Table 3, the potential fulfilment of the DVR recommendations for an average consumption of 1 serving (i.e., 100 g cooked rice) per day was calculated (Table 4), and the average ratio of element concentration in rice for brown to white and for parboiled rice to white rice was calculated and presented in Figure 2, where a ratio above 1 indicates an enrichment of a given element compared to white rice.

**Figure 3:**
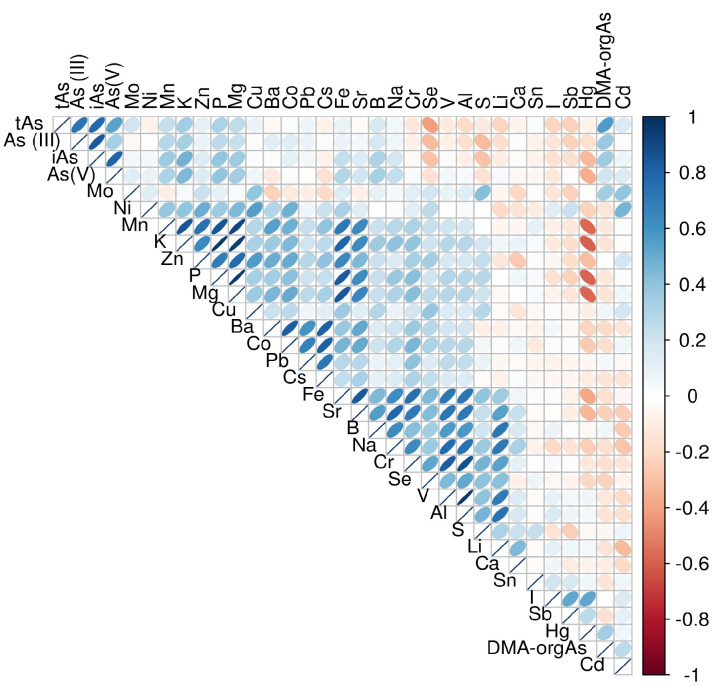
Correlation plot of all analysed elements (a narrow ellipse indicates a strong correlation, while a circular ellipse indicates a weak or no correlation; if the ellipses are tilted to the right, there is a positive correlation, and to the left, a negative correlation).

The correlations for all analysed elements in rice can be found in the correlation diagram (Figure 3).

### 3.3. Composition of the Elements Depending on the Type of Cultivation

**Figure 4:**
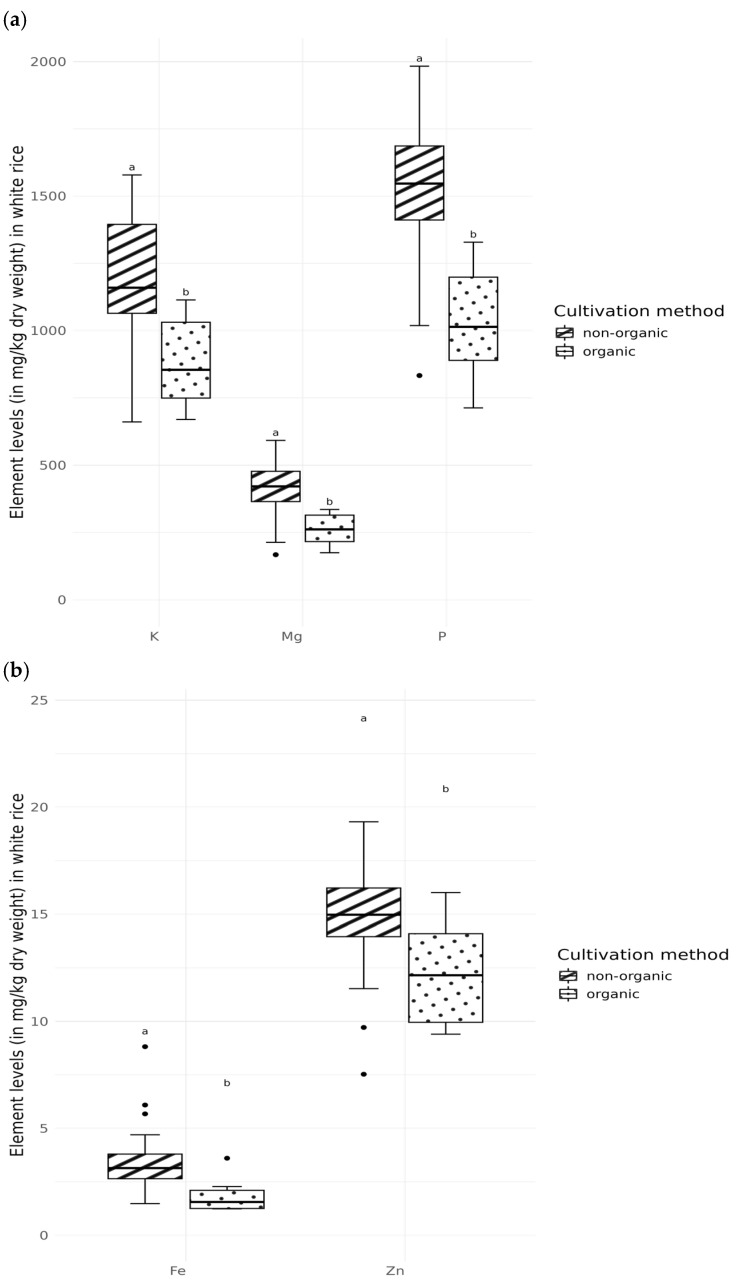
Elements—potassium (K), magnesium (Mg), phosphorus (P) (**a**), iron (Fe), and zinc (Zn) (**b**) (in mg/kg dry weight) in white rice of different cultivation types. The results are presented as box-and-whisker plots, with the whiskers ranging from the minimum to the maximum, the black squares representing the medians, and the dots indicating outliers. Different lowercase letters indicate significant differences (*p* < 0.05), which were tested using the Mann–Whitney U-test.

**Figure 5:**
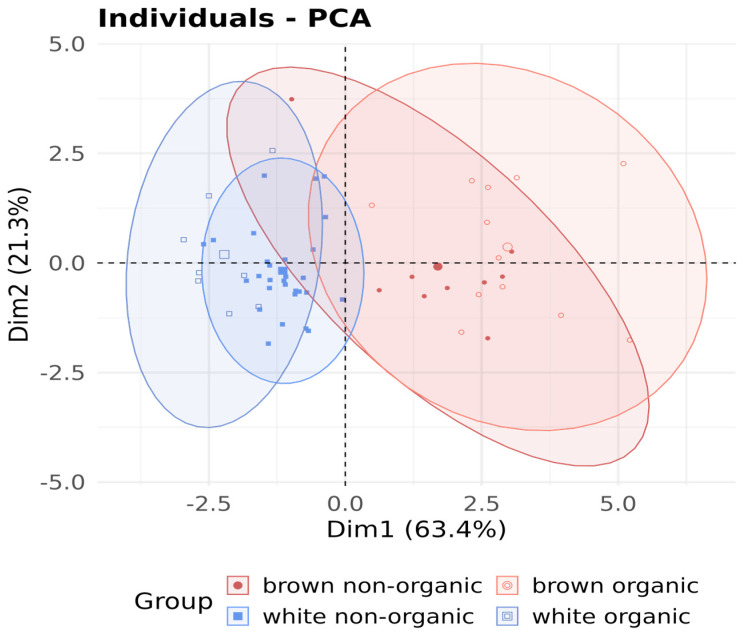
PCA plot of P, K, Mg, Fe, Zn, Se, and tAs separated according to the rice type and origin. The plot shows PC1 and PC2 components.

In addition to the difference between white and brown rice, it is also important to consider whether there is a difference in the composition of mineral elements, depending on the cultivation method, i.e., organic or conventional. In the white rice group, only K, Mg, P, Fe, and Zn differed between non-organic and organic grown samples (Figure 4a,b). For the brown rice group, we found a difference only in Cu (2.66 mg/kg in non-organic vs. 2.03 mg/kg in organic; *p* = 0.0086). Figure 5 shows the principal component analysis (PCA) of the separation depending on the cultivation method and rice variety.

### 3.4. Correlation Between Arsenic and Selenium

**Figure 6:**
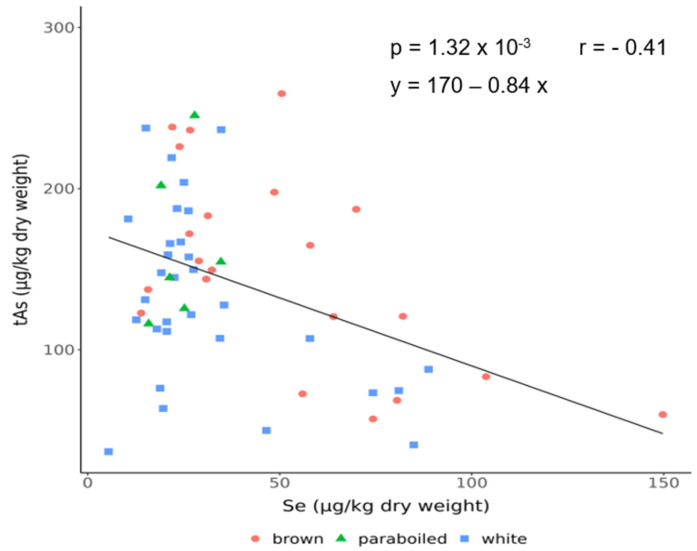
Relationship between the total arsenic (tAs) and selenium (Se) concentrations in analysed rice samples.

As there are studies showing a protective effect of selenium against arsenic toxicity, the relationship between Se and As was investigated. Figure 6 shows the relationship between tAs and Se concentration in the analysed rice samples (*n* = 58) from the Croatian market, and it can be seen that tAs is negatively correlated with Se.

## 4. Discussion

### 4.1. Arsenic in Rice

Chronic exposure to arsenic can have a negative impact on human health and cause many diseases. Most arsenic comes from contaminated drinking water and the environment [28], but it can also be ingested through food. Not only is it present in the same staple foods, but its accumulation in rice can reduce levels of the essential minerals Mn, Ni, and Se [29]. Since it is known that arsenic, like the essential elements, tends to accumulate in the outer parts of the grain, which are removed during polishing, it is to be expected that the arsenic content is higher in brown rice and significantly lower in white rice. The lowest mean tAs concentrations were found in white rice (132.3 µg/kg) followed by brown rice (150.3 µg/kg), and the highest value was in the parboiled rice group (164.7 µg/kg) (Table 1). The observed mean tAs concentrations were within a global “normal” range of 80 to 200 µg/kg for As based on the analysis of more than 200 rice samples from the USA, Europe, Asia, and South America [30]. Each rice group analysed in this study contained some samples with high tAs concentrations. In the white rice group, the highest tAs concentrations were found in two samples from Italy (245 and 238 µg/kg) and in one sample of undetermined EU origin (236 µg/kg), while in the brown and parboiled rice group, two samples of Italian origin had tA concentrations above 200 µg/kg. The highest tAs concentration (258 µg/kg) was found in brown rice of Indian origin. A wide range of tAs concentrations in rice samples has been reported in the literature [15,30]. In Europe, the mean tA concentrations observed ranged from 157 µg/kg in rice purchased on the Slovenian market [5] to 280 µg/kg in various rice varieties grown in Italy [24].

According to Rahman et al. [21], the parboiling process increases the tAs concentration in rice by translocating As from the husk and bran to the kernel of the grain. Although we found no significant differences in tAs by rice type, parboiled rice still had the highest mean tAs value of 164.7 µg/kg (range: 116.1–245.3 µg/kg). At this point, the limitation of the research results due to the small number of samples of parboiled rice (*n* = 7) compared to the other groups observed must be emphasized.

Since more than 70% of the white rice samples were of Italian origin, the differences in tAs by origin were only analysed in the brown rice group. Thus, brown rice from Europe (179 µg/kg) showed twice as high tAs values as samples from Asia (77.9 µg/kg). Similar to our results, Zavala and Duxbury [30] reported twice as high tAs levels in rice grown in Europe (Italy and Spain) (198 µg/kg) compared to Asia (70 µg/kg). With regard to the cultivation method, there were no differences in tAs between conventionally and organically produced rice. Similar results were reported by Menon et al. [23] on tAs in rice samples available on the UK market.

The mean iAs concentration, expressed as the sum of As^III^ and As^V^, was 89.3.5 µg/kg (range: 8.1–200.0) in all rice samples. On average, the iAs concentration was 62.8 ± 22.1% of the tAs concentration in the rice (Table 1). All analysed rice samples were below the EU limit values for inorganic arsenic (iAs): 150 µg/kg for white rice and 250 µg/kg for parboiled rice [31]. Due to its neutral flavour, low allergenic potential, and high nutrient content, rice is one of the most important sources of carbohydrates for infants. Its porridge is often used as the basis for complex meals for children at the beginning of complementary feeding (from 6 months), either in the form of homemade mixtures or commercial products [32]. Although the samples analysed were not classified as rice products for infants, most samples of parboiled rice (5 out of 7) and brown rice (11 out of 20) had iAs concentrations above the limit of 100 µg/kg set for iAs in infant formula rice as prescribed by the European Commission [31], so it is necessary to monitor consumption in infants. Furthermore, the type of rice significantly influenced the As^V^ concentrations. Parboiled rice (54.5 µg/kg) and brown rice (44.3 µg/kg) had twice as high As^V^ concentrations as white rice (22.9 µg/kg). In this study, iAs showed a strong correlation with tAs (r = 0.78, *p* < 0.0001) regardless of whether it was the entire sample or whether it was observed in relation to the type of rice (white, brown, parboiled) (Figure 1). Oteiza et al. [33] reported a moderate iAs/tAs correlation (r = 0.493) between different Argentinian rice varieties. This could be due to the different proportion of iAs in tAs concentrations between rice varieties observed in this study compared to the study conducted in Argentina (62.8% vs. 26.7%). Regarding the oAs species, the MMA concentrations were below 5 µg/kg in all samples, while the mean DMM was 33.0 µg/kg (range: 3.50–170). The lowest mean DMA was found in brown rice (25.6 µg/kg), the next highest mean in parboiled rice (29.2 µg/kg), and the highest in the white rice group (38.7 µg/kg). The proportion of oAs, represented as the sum of DMA and MMA, was 23.2 ± 9.5% of the tAs concentrations in all rice varieties. According to the rice classification based on the predominant As form in rice [34], most of the analysed rice samples in this study belonged to the As^III^ rice type, and only a few samples to the less common DMA rice type (in four samples, DMA accounted for >50% of the tAs).

The estimated intake of iAs (as the sum of As^III^ and As^V^) from a serving portion of different rice types (white, parboiled, and brown) from the Croatian market was compared with the reference values proposed by EFSA [25] and FAO/WHO JECFA [26] (Table 2). The average estimated intake of iAs from one portion was between 0.034 and 0.055 for adults. Taking into account the average consumption rate of rice in the Croatian adult population of 5.1 kg per capita in 2021, which corresponds to about 4 g per day of uncooked rice, the average iAs intake of Croatian adult consumers was 0.004 to 0.007 µg/kg body weight per day, which is well below the dietary exposure estimated by EFSA [25] reported for rice-only consumers (0.01–0.06 µg/kg body weight per day). The average contribution of inorganic arsenic (iAs) in a portion of uncooked rice (33 g for an adult) was estimated between 56.1% (white rice) and 91.9% (for parboiled rice) of the recently established EU reference point of 0.06 µg/kg body weight per day [25].

### 4.2. Dietary Macro and Trace Elements in Rice

The concentrations of 12 different elements quantified by ICP-MS in this study are listed in Table 3. All elements listed in Table 3 differed significantly in their concentrations among the rice varieties (*p* < 0.05) except Mo. Compared to white rice, brown rice had ~3-fold higher concentrations of Mg, Fe, Mn, and Co; twice as high concentrations of P and K; and ~1.5-fold higher concentrations of Ca, Cu, and Se (Figure 2). The observed differences in element concentrations between white and brown rice are consistent with previous studies [4,5,20,33,35]. Moreover, the concentrations of K, Mg, and Zn in the Italian white rice subgroup were comparable to those reported by Sommella et al. [24] on elemental composition in 101 commercial rice samples of Italian origin. Recently, Jo and Todorov [20] found that polishing reduced the content of P, K, Mn, and Fe in brown rice by ~50% and of Cu and Zn by 20%. We observed similar elemental losses depending on the rice variety (Figure 2), with the highest average ratio of brown to white elements being 3 for Mn and 3.5 for Fe and Mg. This is consistent with the data reported by de Oliveira et al. [36] on elemental composition in different Brazilian rice genotypes. Other studies reported a reduction of 16 to 97.4% for Fe and more than 50% for Mn in rice after polishing [27,29]. Da Silva et al. [2] summarized literature data on elemental composition in white, parboiled, and brown rice analysed in the period from 2007 to 2017. Considering these data and the values from more recent studies [4,5,20,27,36,37] on higher Mg, K, Fe, and Mn concentrations in brown rice than in white rice, brown rice can be recommended as a better source of minerals, especially for people suffering from micronutrient deficiencies. The nutrient content of a serving of rice in terms of essential elements was calculated based on the average element concentration (mg/kg) in different rice varieties and considering 33 g of uncooked rice as one serving (equivalent to 100 g of cooked rice). For each element, the estimated intake per serving was expressed as a percentage of the Dietary Reference Value (DRV) for adults established by EFSA [27] (Table 4). According to the present assessment of the percent DRV, all rice varieties are a good source of Mo (33.2%), Mn (19.3%), and P (11.8%). For the other analysed elements, rice can provide between 0.5% and 12% of the DRV. As already mentioned, brown rice is the richest source of dietary trace elements among the analysed rice varieties.

Figure 3 shows the correlation matrix of the analysed elements together with the arsenic specificities in the rice samples, presented as a correlogram. The upper triangle of the correlation matrix with ellipses indicates the strength and direction of the correlation. Blue stands for a positive correlation (the darker, the stronger), red for a negative one, while the beige/light colours indicate a weak or no correlation. Total As, As^III^, As^V^, and iAs are, as shown before, strongly positively correlated with each other, which is to be expected as they are all forms of the same element, and this is consistent with findings from recent studies [38]. In addition, a positive correlation was observed between Mn, Fe, Co, Zn, Ni, and Cu, indicating that these elements can accumulate in plants. Na, B, Fe, Cr, and Se also formed a group with a strong positive correlation to each other.

### 4.3. Composition of the Elements Depending on the Type of Cultivation

**Figure A1:**
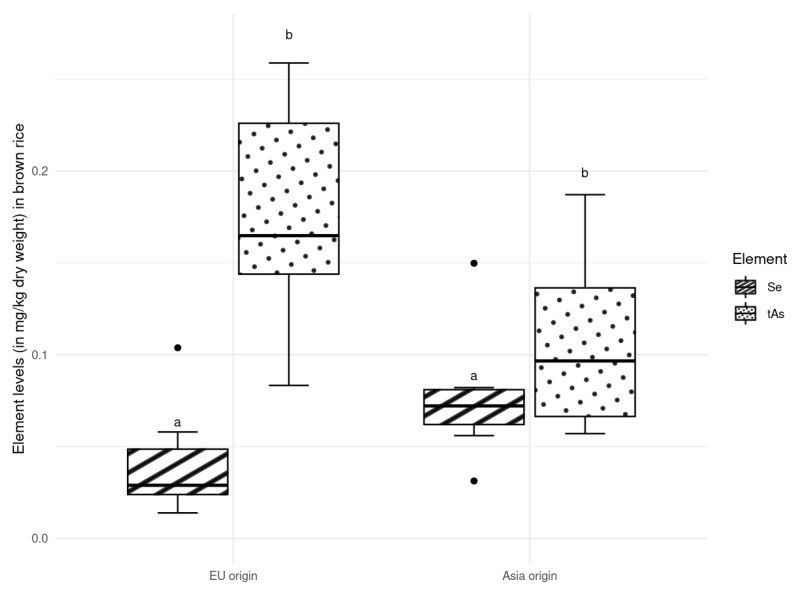
Total arsenic (tAs) and selenium levels (Se) (mg/kg dry weight) in brown rice samples of different origins. The results are shown as box-and-whisker plots, with whiskers from minimum to maximum, black squares as medians, dots as outliers, and asterisks as extremes. Different lowercase letters indicate significant differences (at *p* < 0.05), which were tested using the Mann–Whitney U-test.

In addition to the difference between white and brown rice, we analysed the effects of different cultivation methods and origin on the essential element concentrations in rice. In the white rice group, the conventionally produced (non-organic) samples had two times higher P, K, Mg, Fe, and Zn concentrations compared to organically grown samples (Figure 4a,b), while in the brown rice group, the samples varied only in Cu (2.66 vs. 2.03 mg/kg, respectively; *p* = 0.0086). When we summarized the data of all rice varieties, there were no significant differences in element concentrations between conventionally and organically grown rice. Regarding the origin, rice from the EU had significantly lower Fe, Co, Cu, and Se values than rice from Asia (at *p* < 0.01). Since 85% of the white rice samples were from Italy, we did not test for any differences based on origin in this group. Among the brown rice group, only the Se concentrations differed between Asian (0.083 mg/kg) and EU rice (0.032 mg/kg) (Figure A1 in the Appendix A).

**Figure A2:**
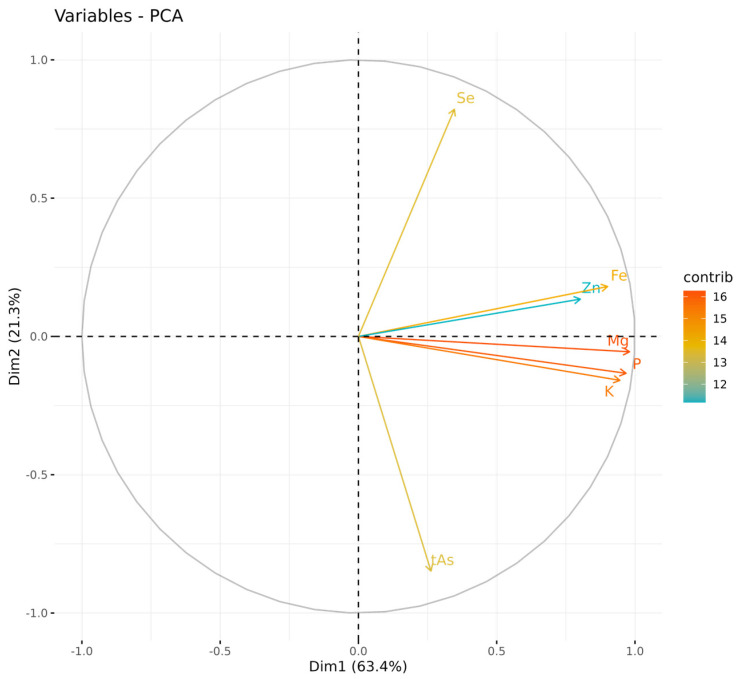
PCA loading plot. Plot displays the projection of each variable as a vector within the correlation circle defined by the first two principal components. The direction and length of each arrow indicate the strength and orientation of the variable’s contribution to the principal components, while the colour gradient reflects the magnitude of each variable’s contribution. Variables with similar vector directions are positively correlated, while those oriented in opposite directions are negatively correlated.

Principal component analysis (PCA) was performed to evaluate the influence of cultivation method and rice variety on the elemental composition of rice grains. The PCA visualization revealed clear grouping patterns corresponding to both the cultivation method and the rice variety. The results show a clear separation between white and brown rice along the first principal component (explaining 63.4% of the variance) according to the observed elements (P, K, Mg, Fe, Zn, Se, tAs). This is consistent with previous findings that processing (milling) significantly reduces the mineral content of white rice [39,40]. The PCA loading plot (Figure A2) shows the projection of each variable as a vector within the circle of correlation defined by the first two principal components, which together explain 84.7% of the total variance. It is noteworthy that K, P, and Mg have the highest contributions to Dim1, suggesting that they are the most influential elements in explaining the variance captured by the first two components. Brown rice, which retains the aleurone layer and germ, is rich in minerals, whereas white rice is polished and loses many of these nutrients during milling. Studies have consistently shown that brown rice has a significantly higher content of P, K, Mg, Fe, and Zn compared to white rice [23,41]. The second component (explaining 21.3% of the variance) reflects the variability associated with the farming system. Organically grown brown and white rice can be separated from each other, while there is overlap in non-organically grown rice. A similar pattern has been observed in other studies showing higher concentrations of micronutrients (especially Zn and Fe) in organically grown cereals and pulses [42,43].

### 4.4. Correlation Between Arsenic and Selenium

Regarding the correlation with As, we found only one significant correlation between tAs and Se among all analysed elements in rice (r = −0.41, *p* = 0.0013) (Figure 6). It can be seen that tAs is negatively correlated with Se, but this correlation may not be so significant if all analysed rice samples are correlated (the correlation coefficient r was not higher than 0.5). When the correlation within a particular rice variety is taken into account, there is a significantly higher correlation between the subgroups of brown rice (r = −0.6). It is important to note that EU brown rice has a 4-fold higher As:Se ratio (5.7) than Asian brown rice (1.4). Šlejkovec et al. [5] also reported a negative As:Se relationship in different rice varieties from the Slovenian market with a similar r range between varieties (from −0.30 to −0.60). High As concentrations in rice are associated with low concentrations of the essential element Se, which may lead to an increased health risk due to toxic As exposure and Se deficiency. Soil biogeochemistry and site effects (such as As-contaminated groundwater) have a greater influence on the As and Se content in the grain than the rice variety or genotype [12,15,24,28]. In contrast to As and Se, trace elements such as Co, Cu, Fe, Mn, and Zn in rice are genotype-dependent [12].

## 5. Conclusions

In the present study, tAs concentrations in the rice samples ranged from 36.7 to 259.0 µg/kg dry weight, with the lowest average tAs values in the white rice samples (132.3 µg/kg), followed by brown rice (150.3 µg/kg), and with the highest values in the par-boiled rice group (164.7 µg/kg). Although some samples showed high levels of tAs (up to 259 µg/kg), none of the analysed samples exceeded the limit value for iAs in rice set by the European Commission [31]. Except for Mo, all dietary macro and trace elements analysed in rice differed considerably depending on the rice variety. In the brown rice group, the levels of P, K, Mg, Fe, and Zn were influenced by the cultivation method, while the levels of tAs and Se were influenced by the respective other levels and the origin. Overall, brown rice has been shown to be a better source of essential elements compared to white rice, especially for Mg, Mn, Fe, and Co. However, due to the higher accumulation of arsenic species, caution should be exercised when including brown rice in the diet of sensitive groups such as young children. These results highlight the importance of balancing the nutritional benefits against the potential toxicological risks and suggest that further research is needed to optimize cultivation and processing.

## Figures and Tables

**Table 2:** The average contribution (as a percentage of the reference value) of inorganic arsenic (iAs) in one serving of white, parboiled, and brown rice available on the Croatian market for an adult person of 70.8 kg.

	White Rice	Parboiled Rice	Brown Rice
EI (µg/kg bw)	0.034	0.055	0.049
% RP of 0.06 µg/kg bw/day [25]	56.1	91.9	82.3
% BDML_05_ of 3 µg/kg bw/day [26]	1.1	1.8	1.6

EI: estimated intake per serving in µg/kg bw. The EI was calculated according to the following formula: EI_iAs_ = (C_iAs_ × MS)/BW, where C represents the concentration of iAs [μg/kg d.w.], MS denotes a serving size of 33 g of uncooked rice grains for adults that corresponds to 100 g of cooked rice, and BW is the average adult body weight of 70.8 kg. The determined EIs were compared with the reference values established by the EFSA [25] and the JECFA [26]. BMDL: benchmark dose lower confidence limit. RP: reference point.

**Table 3:** Dietary macro and trace element concentrations (mg/kg dry weight) in white, parboiled, and brown rice available on the Croatian market and in standard reference material, SRM 1568b rice flour (NIST, Gaithersburg, MD, USA).

Element (mg/kg)	All Rice (*n* = 58)	White Rice (*n* = 31)	Parboiled Rice (*n* = 7)	Brown Rice (*n* = 20)	SRM 1568b (*n* = 4)
	Mean ± SD (Range)	Mean ± SD (Range)	Mean ± SD (Range)	Mean ± SD (Range)	Mean ± SD [Mean ± 95% CI]
Mg	705 ± 482 (168–1931)	369 ± 115 ^a^ (168–592)	450 ± 75 ^a^ (325–514)	1274 ± 330 ^b^ (438–1931)	539 ± 3 [559 ± 10]
P	1959 ± 811 (712–4281)	1372 ± 322 ^a^ (713–1983)	1896 ± 245 ^b^ (1583–2116)	2846 ± 585 ^c^ (1365–4281)	1515 ± 5 [1530 ± 40]
K	1732 ± 818 (661–3913)	1103 ± 262 ^a^ (661–1579)	1866 ± 186 ^b^ (1584–2078)	2624 ± 585 ^c^ (1132–3913)	1130 ± 6 [1282 ± 11]
Ca	114.2 ± 179.4 (36.2–1144.1)	91.9 ± 155.5 ^a^ (36.2–912.1)	234.4 ± 445.8 ^a^ (37.4–1144.1)	112.7 ± 25.2 ^b^ (61.1–156.7)	112 ± 2 [118.4 ± 3.1]
Fe	6.06 ± 4.91 (1.24–24.43)	3.06 ± 1.64 ^a^ (1.24–8.82)	3.43 ± 0.29 ^a^ (2.95–3.76)	11.3 ± 4.53 ^b^ (4.04–24.43)	7.05 ± 0.06 [7.42 ± 0.44]
Mn	17.6 ± 11.5 (4.78–43.6)	10.06 ± 3.10 ^a^ (4.78–17.46)	10.39 ± 3.86 ^a^ (6.35–17.72)	30.64 ± 8.78 ^b^ (10.75–43.64)	18.45 ± 0.03 [19.2 ± 1.8]
Co	0.011 ± 0.012 (<LoD–0.072)	0.006 ± 0.003 ^a^ (0.001–0.016)	0.006 ± 0.003 ^a^ (0.003–0.013)	0.019 ± 0.017 ^b^ (0.004–0.072)	0.0223 ± 0.0004 [0.0177 ± 0.0005]
Cu	2.11 ± 0.661 (0.932–3.871)	1.99 ± 0.723 ^a^ (0.932–3.868)	1.79 ± 0.421 ^a^ (1.38–2.40)	2.39 ± 0.527 ^b^ (1.35–3.24)	2.29 ± 0.02 [2.35 ± 0.16]
Zn	15.6 ± 4.10 (7.52–22.81)	14.1 ± 2.81 ^a^ (7.53–19.32)	10.2 ± 2.14 ^b^ (7.52–13.70)	19.1 ± 3.06 ^c^ (11.64–22.76)	20.09 ± 0.13 [19.42 ± 0.26]
Se	0.038 ± 0.028 (<LoD–0.151)	0.032 ± 0.022 ^a^ (<LoD–0.089)	0.024 ± 0.007 ^ab^ (0.016–0.035)	0.052 ± 0.034 ^b^ (0.014–0.150)	0.335 ± 0.035 [0.365 ± 0.029]
Mo	0.654 ± 0.181 (0.339–1.197)	0.665 ± 0.203 (0.339–1.200)	0.624 ± 0.127 (0.418–0.768)	0.647 ± 0.164 (0.390–1.012)	1.430 ± 0.008 [1.451 ± 0.048]

Elements are listed in order of increasing atomic mass. LoD—limit of detection (µg/kg): Mg: 0.003; P: 0.013; K: 0.046; Ca: 0.024; Fe: 0.008; Mn: 0.00002; Co: 0.0021; Cu: 0.0002; Zn: 0.0004; Se: 0.010; Mo: 0.00004. Horizontally, different lowercase letters denote significant differences (at *p* < 0.05) tested by Kruskal–Wallis ANOVA and post-hoc pairwise multiple comparison of mean ranks for three groups (white, parboiled, and brown rice) using Dunn’s test.

**Table 4:** Contribution of a serving portion of different types of rice in relation to the essential elements.

Element	DRV (mg/day)	Estimated Intake per Serving (% DRV)			
		All Rice	White Rice	Parboiled Rice	Brown Rice
Mg	300–**350**	6.6	3.5	4.2	12.0
P	550	11.8	8.2	11.4	17.1
K	3500	1.6	1.0	1.8	2.5
Ca	1000	0.4	0.3	0.8	0.4
Fe	11	1.8	0.9	1.1	3.4
Mn	3	19.3	11.1	11.4	33.7
Cu	1.3–**1.6**	4.4	4.1	3.7	4.9
Zn	7.5–**16.3**	3.1	2.9	2.1	3.9
Se	0.07	1.8	1.5	1.1	2.5
Mo	0.065	33.2	33.8	31.7	32.8

DRV (bold)—dietary reference values [27] for adults used to calculate the % DRV. The serving portion is estimated at 33 g of uncooked rice grains as equivalent to 100 g of cooked rice grains.

## Data Availability

The original contributions presented in this study are included in the article/supplementary material. Further inquiries can be directed to the corresponding authors.

## Supplementary Materials

The following supporting information can be downloaded at: https://www.mdpi.com/article/10.3390/foods14132261/s1, Table S1. Rice types purchased at the Croatian market and analysed in this study; Table S2. Temperature programme for digestion of dry rice samples in the microwave digestion system UltraCLAVE IV (Milestone, Italy).

## Abbreviations

The following abbreviations are used in this manuscript:

ICP-MS	inductively coupled plasma mass spectrometry
HPLC	high-performance liquid chromatography
MMA	monomethylarsonic acid
DMA	dimethylarsinic acid
LoD	limit of detection
EFSA	European Food Safety Authority
WHO	World Health Organization
EU	European Union
DRV	Dietary Reference Value
PCA	Principal Component Analysis

## Appendix A

**Table A1:** Concentrations of non-essential elements (mg/kg dry weight) in white, parboiled, and brown rice available on the Croatian market.

Element	All Rice	White Rice	Parboiled Rice	Brown Rice
(mg/kg)	(n = 58)	(n = 31)	(n = 7)	(n = 20)
	Mean ± SD (Range)	Mean ± SD (Range)	Mean ± SD (Range)	Mean ± SD (Range)
Li	0.013 ± 0.024 (0.003–0.111)	0.012 ± 0.003 (0.003–0.075)	0.014 ± 0.024 (0.003–0.063)	0.014 ± 0.030 (0.003–0.111)
B	0.887 ± 0.363 (0.397–2.514)	0.806 ± 0.272 (0.495–1.697)	0.966 ± 0.332 (0.702–1.599)	0.983 ± 0.465 (0.397–2.514)
Na	12.14 ± 9.35 (3.30 -59.11)	8.44 ± 7.45 ^a^ (3.30–29.35)	16.85± 5.30 ^b^ (12.36–25.81)	16.26 ± 10.62 ^a^ (8.27–59.11)
Al	1.719 ± 2.905 (0.2–18.291)	1.083 ± 1.848 ^a^ (0.2–9.126)	0.789 ± 0.868 ^a^ (0.2–2.491)	2.922 ± 4.042 ^b^ (0.2–18.291)
S	1128.48 ± 202.79 (725.88–1622.56)	1128.81 ± 202.0 (725.88–1584.58)	1122.73 ± 61.45 (1067.76–1242.12)	1129.80 ± 234.62 (751.27–1622.56)
V	0.005 ± 0.006 (0.0023–0.0445)	0.0034 ± 0.0028 ^a^ (0.0023–0.0143)	0.0039 ± 0.0025 ^a^ (0.0023–0.0073)	0.0076 ± 0.0095 ^b^ (0.0023–0.0445)
Cr	0.035 ± 0.049 (0.0045–0.2431)	0.0203 ± 0.0325 ^a^ (0.0045–0.1785)	0.0215 ± 0.0122 ^ab^ (0.0085–0.0444)	0.0616 ± 0.0648 ^b^ (0.014–0.2431)
Ni	0.373 ± 0.289 (0.083–1.402)	0.286 ± 0.221 ^a^ (0.083–0.977)	0.208 ± 0.123 ^a^ (0.112–0.428)	0.550 ± 0.331 ^b^ (0.172–1.402)
Sr	0.272 ± 0.211 (0.062–1.146)	0.154 ± 0.126 ^a^ (0.062–0.558)	0.185 ± 0.073 ^a^ (0.117–0.319)	0.471 ± 0.191 ^b^ (0.214–1.146)
Sn	0.038 ± 0.169 (0.0027–1.03584)	0.0344 ± 0.1426 0.0027–0.7887)	0.0027 ± 0.0 (0.0027–0.0027)	0.0531 ± 0.2252 (0.0027–1.0358)
Sb	0.006 ± 0.011 (0.001–0.068)	0.007 ± 0.014 (0.001–0.068)	0.002 ± 0.001 (0.001–0.004)	0.005 ± 0.005 (0.001–0.019)
I	0.145 ± 0.089 (0.03–0.347)	0.158 ± 00.101 (0.03–0.347)	0.077 ± 0.075 (0.03–0.201)	0.146 ± 0.064 (0.03–0.217)
Cs	0.015 ± 0.057 (0.00003–0.3486)	0.001 ± 0.0021 ^a^ (0.00003–0.0112)	0.005 ± 0.012 ^a b^ (0.00028–0.0294)	0.040 ± 0.091 ^b^ (0.0003–0.3486)
Ba	0.309 ± 0.589 (0.023–2.970)	0.095 ± 0.160 ^a^ (0.023–0.891)	0.081 ± 0.021 ^a^ (0.061–0.108)	0.690 ± 0.844 ^b^ (0.107–2.970)
Hg	0.013 ± 0.008 (0.002–0.031)	0.017 ± 0.006 ^a^ (0.008–0.031)	0.008 ± 0.002 ^b^ (0.007–0.10)	0.007 ± 0.006 ^b^ (0.002–0.026)
Pb	0.006 ± 0.012 (0.003–0.090)	0.003 ± 0.005 (0.003–0.003)	0.003 ± 0.0 (0.003–0.030)	0.009 ± 0.019 (0.003–0.090)
Cd	0.037 ± 0.035 (0.002–0.208)	0.040 ± 0.033 (0.002–0.152)	0.014 ± 0.009 (0.002–0.029)	0.039 ± 0.042 (0.002–0.208)

Horizontally, different lowercase letters denote significant differences (at *p* < 0.05) tested by Kruskal–Wallis ANOVA and post-hoc pairwise multiple comparison of mean ranks for three groups (white, parboiled, and brown rice) using Dunn’s test.
