# Supplementary material for: Dietary Trace Elements and Arsenic Species in Rice: A Study of Samples from Croatian Supermarkets
Source: Foods. 2025 Jun 26;14(13):2261. doi: 10.3390/foods14132261 (PMC12249309; doi:10.3390/foods14132261)
Supplement: Supplementary file 1 [file foods-14-02261-s001.zip › foods-3704693-supplementary.pdf]

Supplementary Material

Table S1. Rice types purchased at the Croatian market and analyzed in this study.

	Rice Type	Cultivation Type	Grain size	Additional Description	Origin
1.	White	Organic	Short	Bio	Italy
2.	White	Organic	Long	Arborio	Italy
3.	White	Organic	Short	Milchreise	Germany
4.	White	Organic	Short	/	Italy
5.	White	Organic	Short	/	Italy
6.	White	Organic	Short	/	Italy
7.	White	Organic	Long	Yasmin	Cambodia
8.	White	Organic	Long	Basmati	India
9.	White	Non-organic	Medium	White	Italy
10.	White	Non-organic	Medium	Arborio	Italy
11.	White	Non-organic	Long	Sant Andrea	Italy
12.	White	Non-organic	/	Arborio	Italy
13.	White	Non-organic	Long	Roma	Italy
14.	White	Non-organic	Long	/	Italy
15.	White	Non-organic	Medium	Sant Andrea	Italy
16.	White	Non-organic	Long	Arborio	Italy
17.	White	Non-organic	Long	/	Italy
18.	White	Non-organic	Long	Basmati	Belgium
19.	White	Non-organic	Long	/	Belgium
20.	White	Non-organic	Long	Basmati	Pakistan
21.	White	Non-organic	Short	/	unknown
22.	White	Non-organic	Long	/	unknown
23.	White	Non-organic	Long	Roma	Italy
24.	White	Non-organic	Short	Originario	Italy
25.	White	Non-organic	Short	Originario	Italy
26.	White	Non-organic	Long	Carnarolli	Italy
27.	White	Non-organic	Long	/	Italy
28.	White	Non-organic	Long	Basmati	Italy
29.	White	Non-organic	/	/	Italy
30.	White	Non-organic	Long	Arborio	Italy

Table S1. *(continued)*

	Rice Type	Cultivation Type	Grain size	Additional Description	Origin
31.	White	Non-organic	Long	/	Europe
32.	Parboiled	Non-organic	Medium	White	Italy
33.	Parboiled	Non-organic	Long	White	Europe
34.	Parboiled	Non-organic	/	Blond	Italy
35.	Parboiled	Non-organic	Long	White	Italy
36.	Parboiled	Non-organic	Long	White	Italy
37.	Parboiled	Non-organic	Long	White	Italy
38.	Brown	Organic	Long	Arborio	Italy
39.	Brown	Organic	Long	Thai	Italy
40.	Brown	Organic	Short	/	Italy
41.	Brown	Organic	Long	Basmati	India
42.	Brown	Organic	Long	/	Europe
43.	Brown	Organic	Long	Basmati	India
44.	Brown	Organic	Long	Naturreis	India
45.	Brown	Organic	Long	Yasmin	Thailand
46.	Brown	Organic	Long	Basmati	Pakistan
47.	Brown	Organic	Short	Sweet	USA
48.	Brown	Organic	Long	Red	Cambodia
49.	Brown	Organic	Long	Black	Thailand
50.	Brown	Non-organic	Long	Black	Italy
51.	Brown	Non-organic	Long	Red	France
52.	Brown	Non-organic	Long	Blond	Italy
53.	Brown	Non-organic	Long	/	Belgium
54.	Brown	Non-organic	Long	Basmatio	India
55.	Brown	Non-organic	Long	Roma	Italy
56.	Brown	Non-organic	Medium	/	unknown
57.	Brown	Non-organic	Long	/	Italy
58.	Parboiled	Non-organic	Medium	Brown	Italy

Table S2. Temperature program for the digestion of dry rice samples in the microwave digestion system UltraCLAVE IV (Milestone, Italy).

	T (min:s)	E (W)	T1 (°C)	T2 (°C)	p (bar)
1.	3:30	700	70	70	100
2.	15	1000	180	70	100
3.	10	1000	250	70	140
4.	30	1000	250	70	140
5.	40	0	30	70	20
